# Supplementary material for: A Chromosome-Level Genome of the Camphor Tree and the Underlying Genetic and Climatic Factors for Its Top-Geoherbalism
Source: Front Plant Sci. 2022 Apr 21;13:827890. doi: 10.3389/fpls.2022.827890 (PMC9112071; doi:10.3389/fpls.2022.827890)
Supplement: Supplementary file 1 [file Presentation_1.PPTX]

## Slide 1
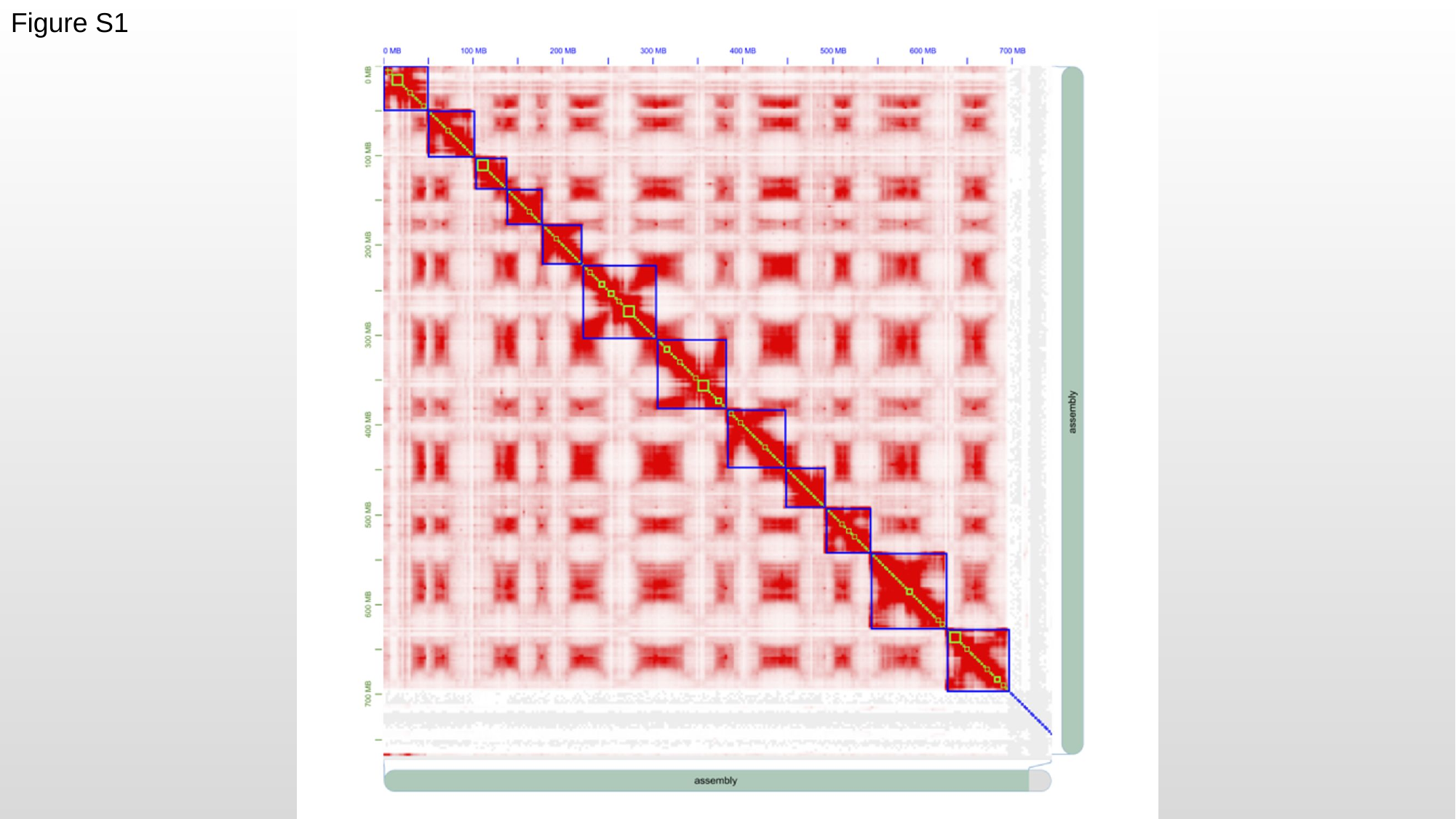

Figure S1

## Slide 2
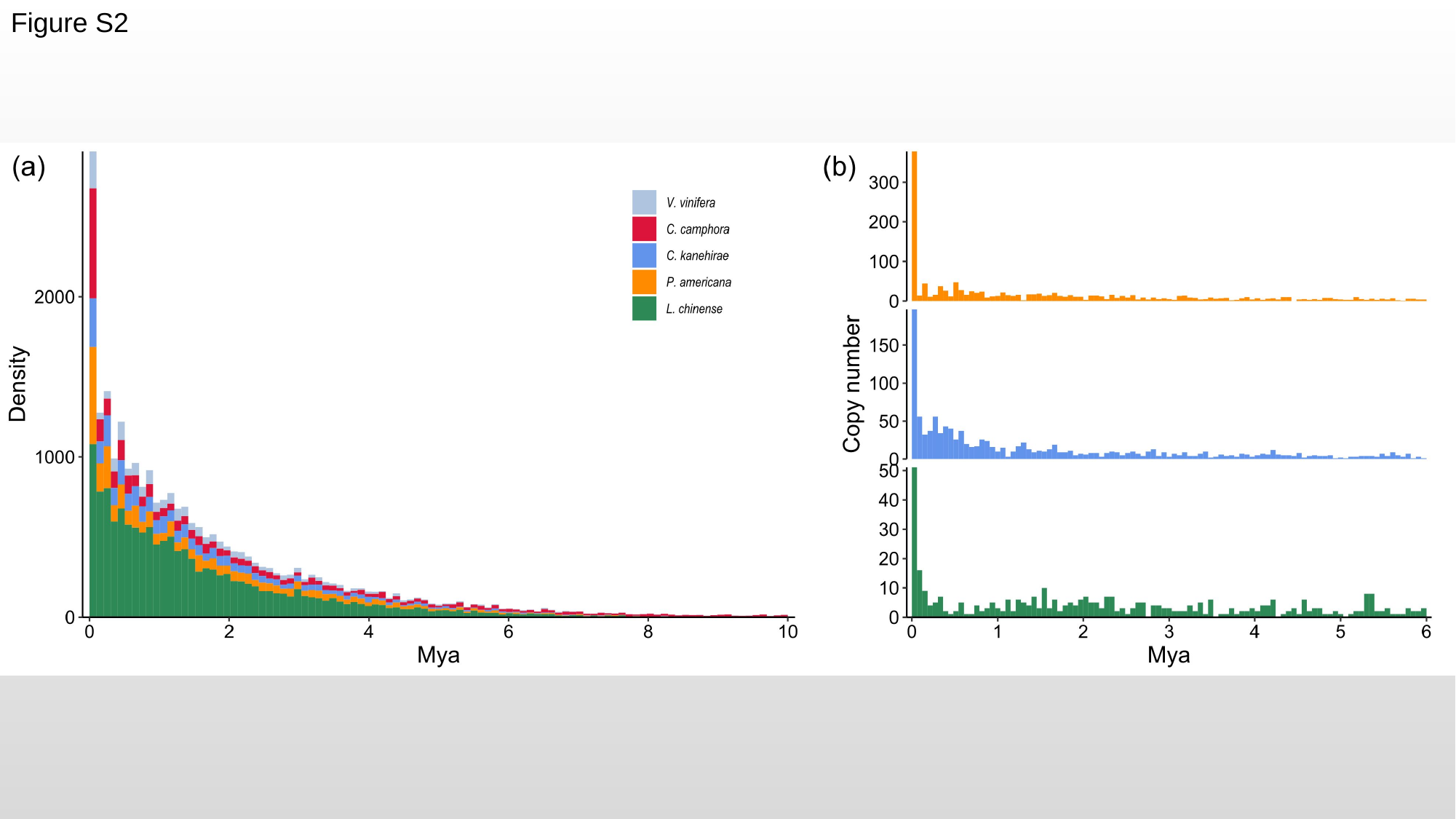

Figure S2

## Slide 3
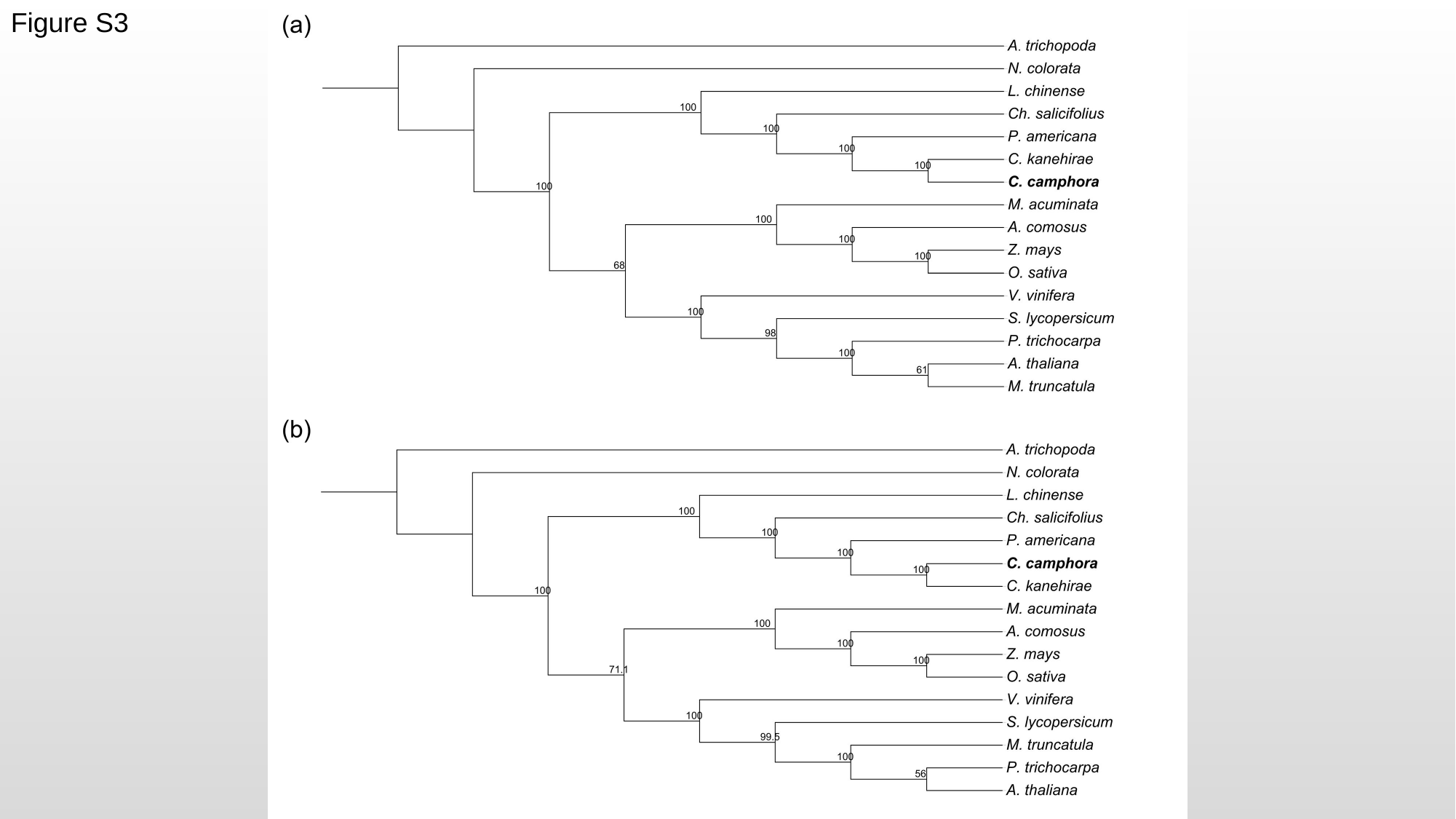

Figure S3

## Slide 4
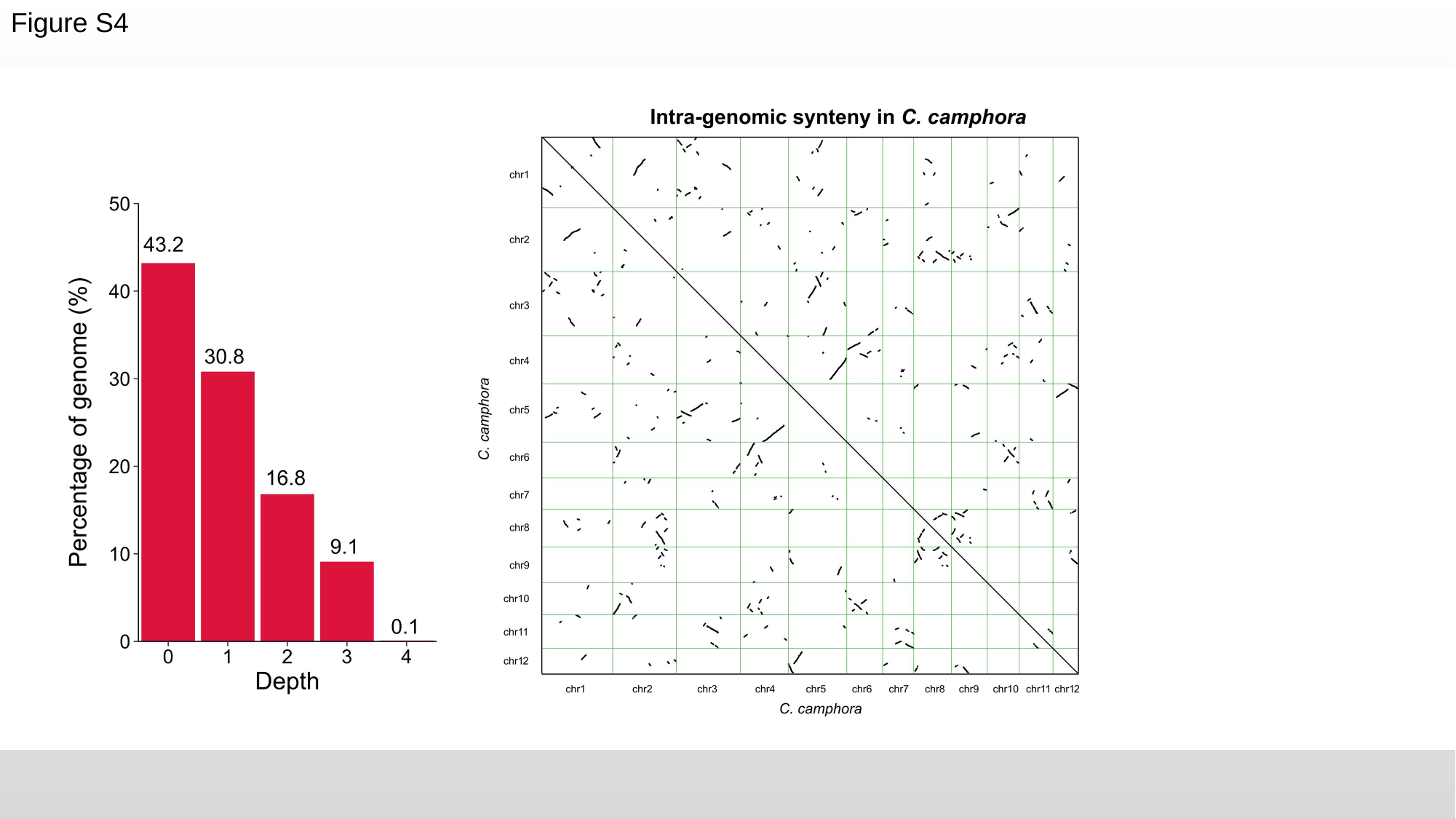

Figure S4

## Slide 5
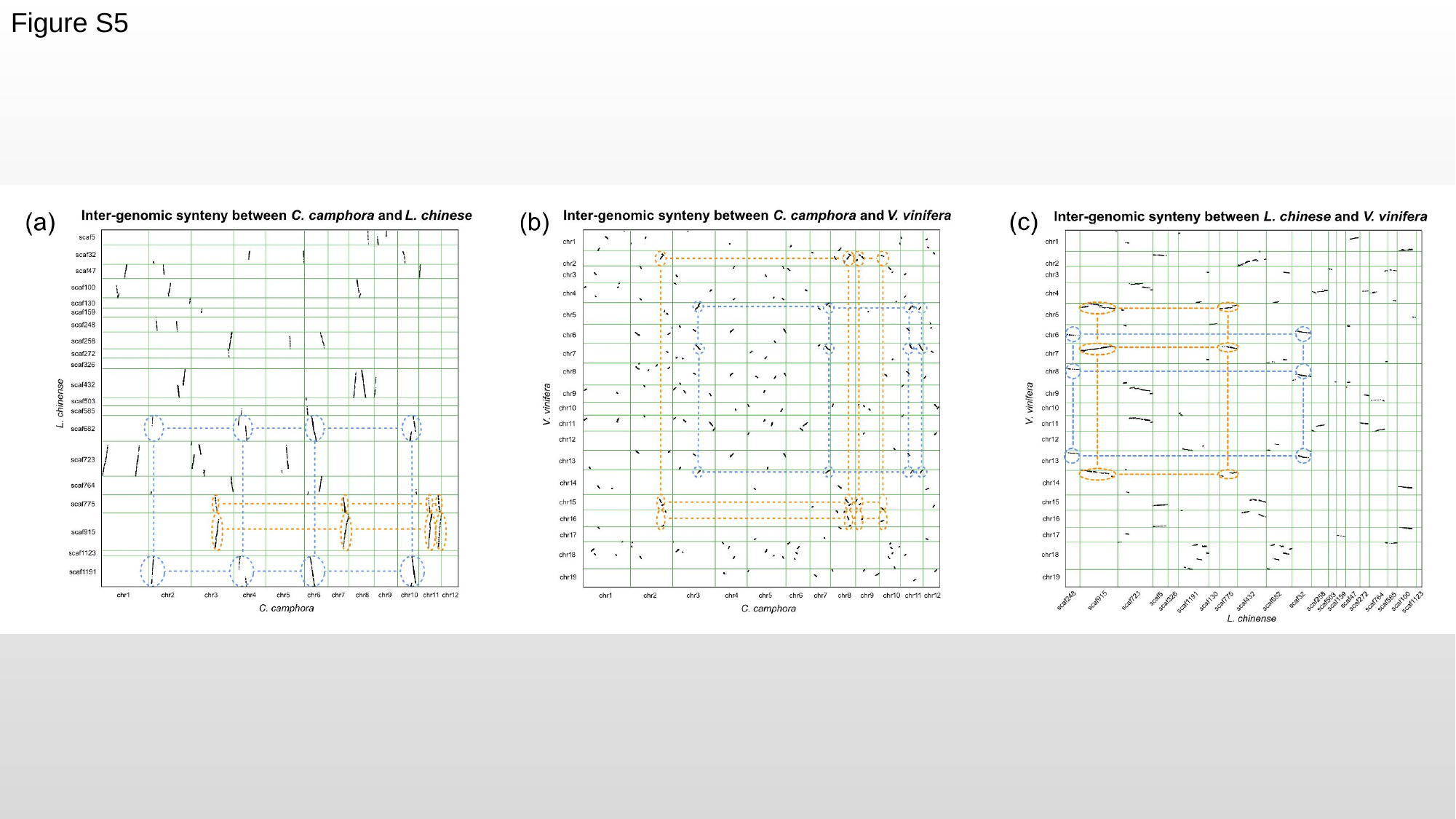

Figure S5

## Slide 6
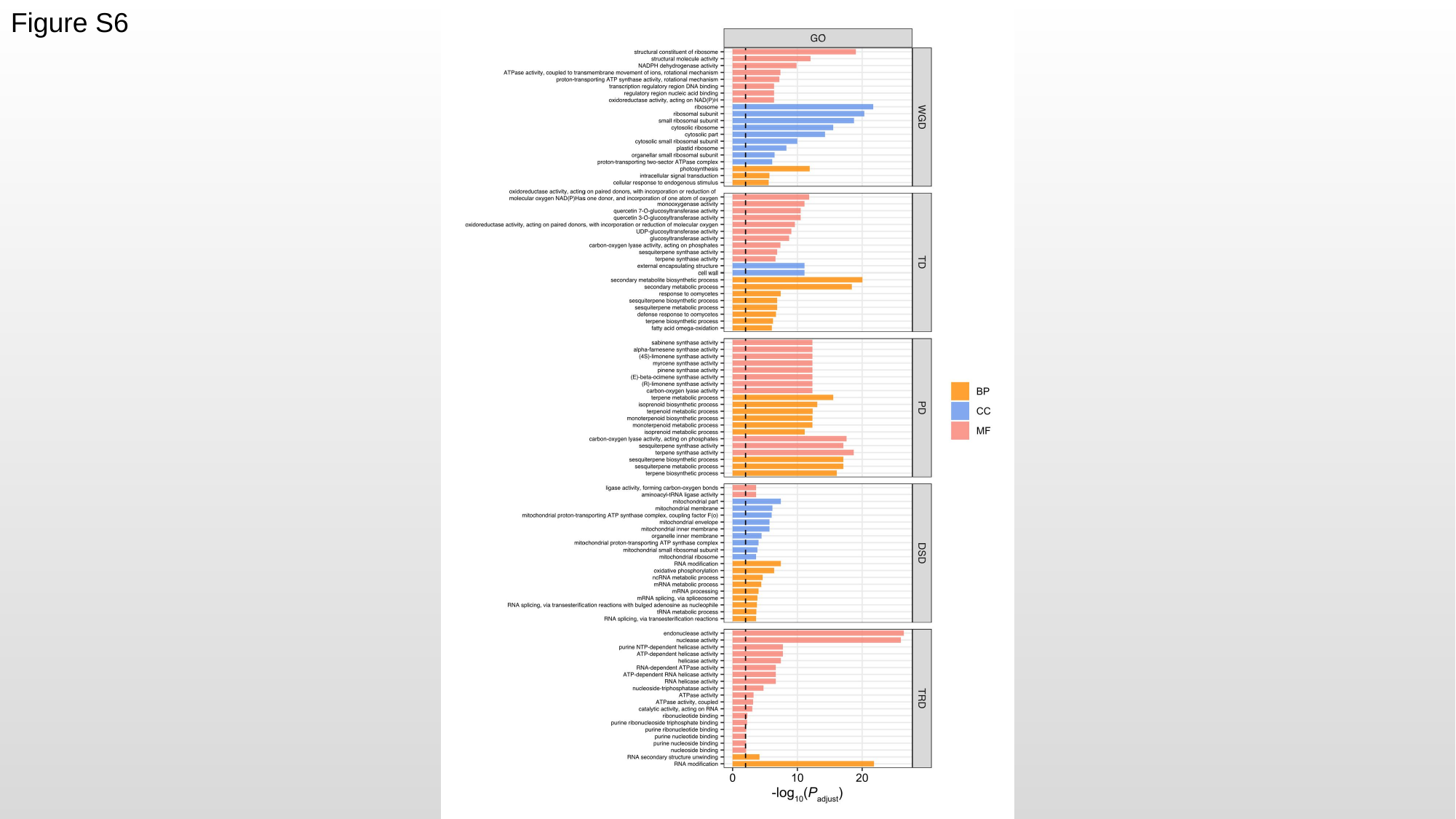

Figure S6

## Slide 7
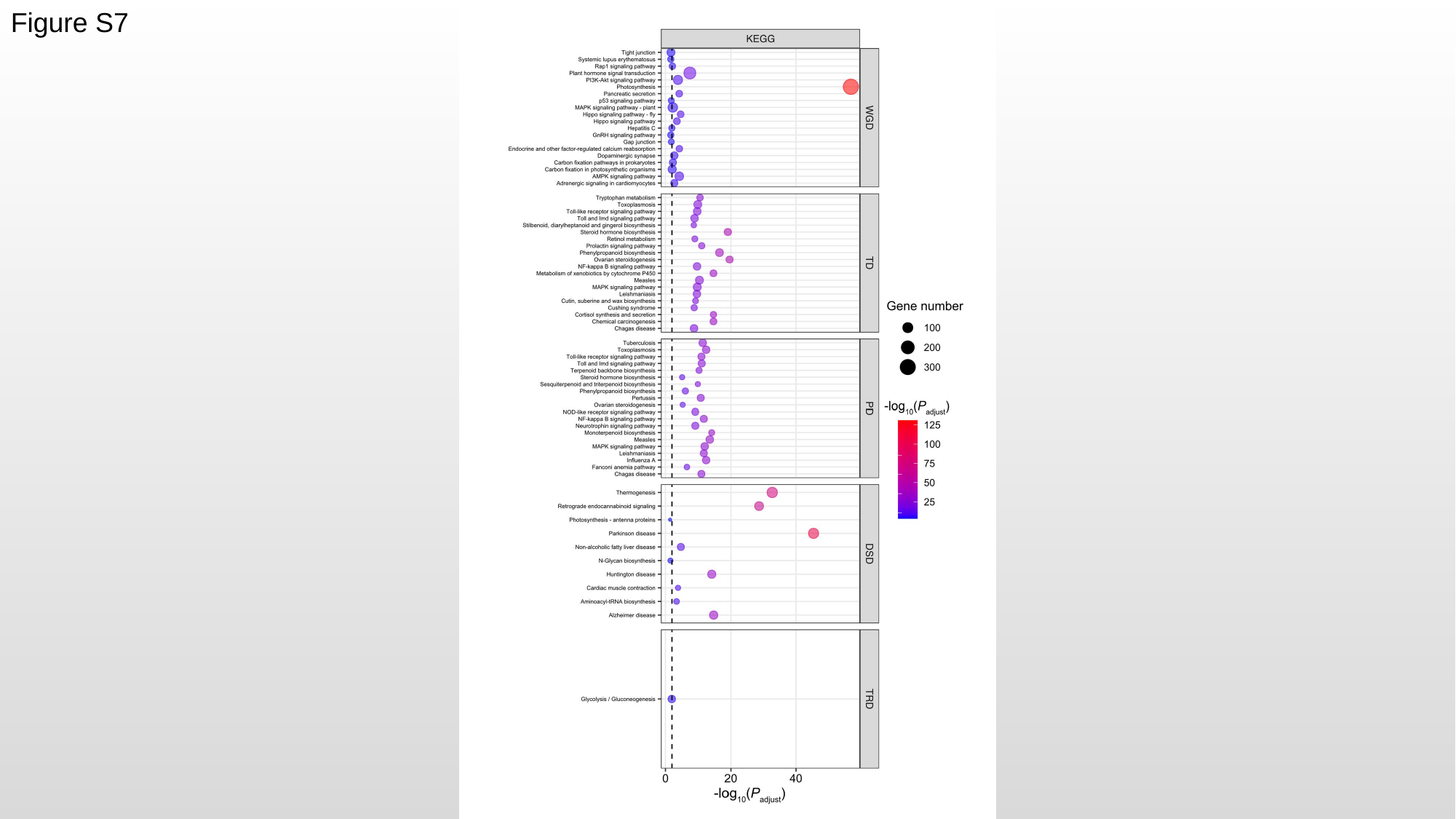

Figure S7

## Slide 8
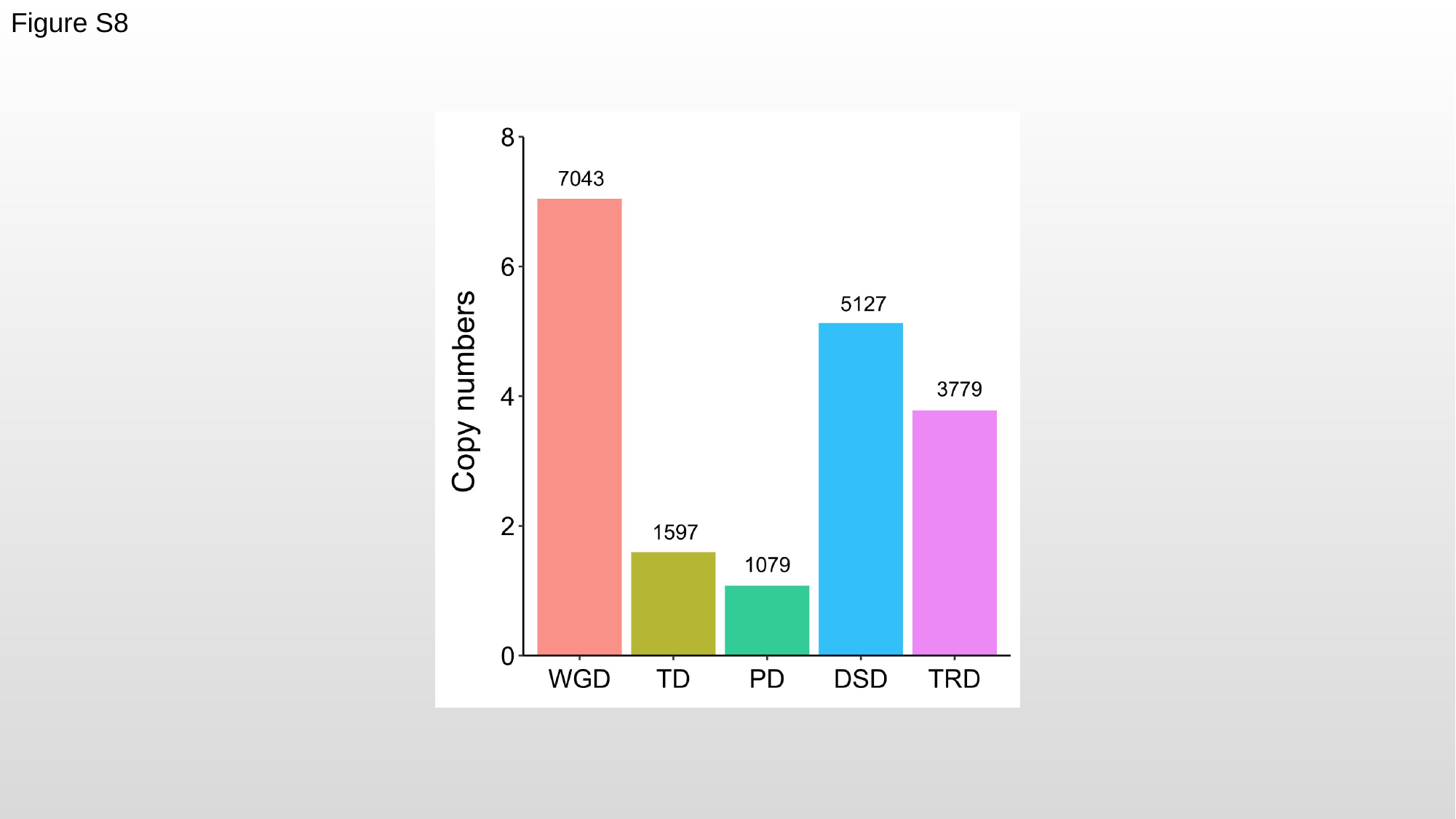

Figure S8

## Slide 9
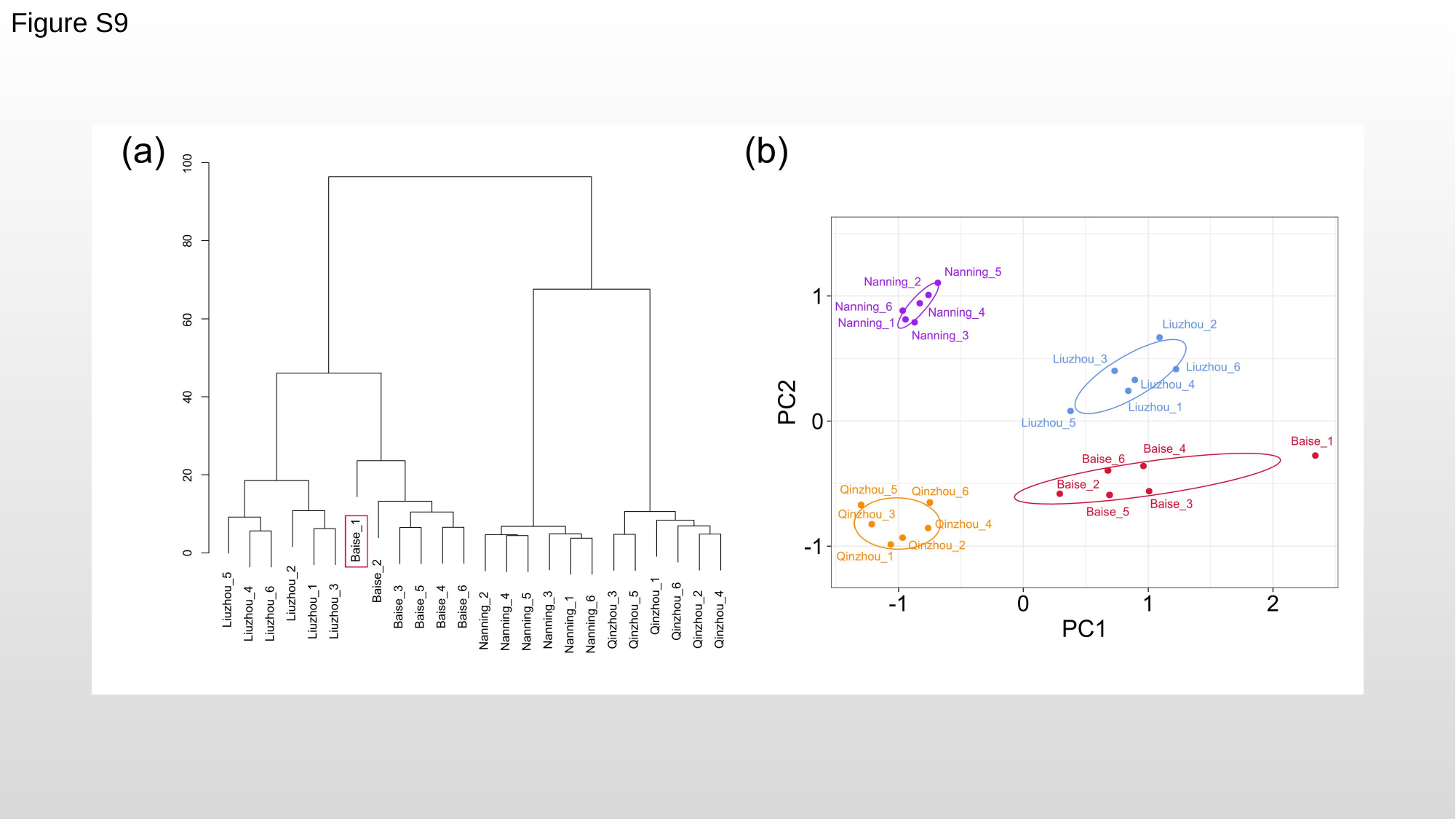

Figure S9

## Slide 10
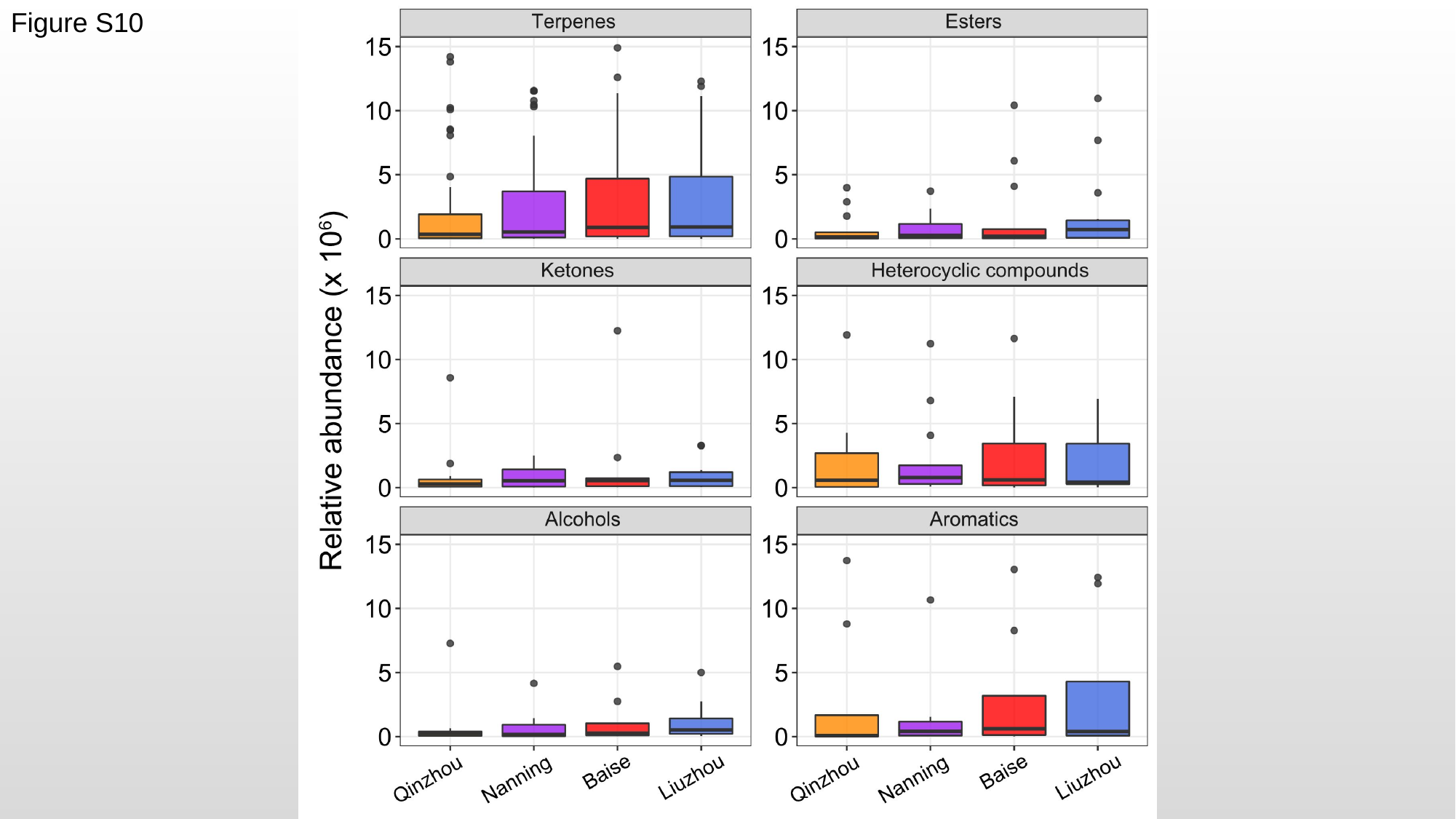

Figure S10

## Slide 11
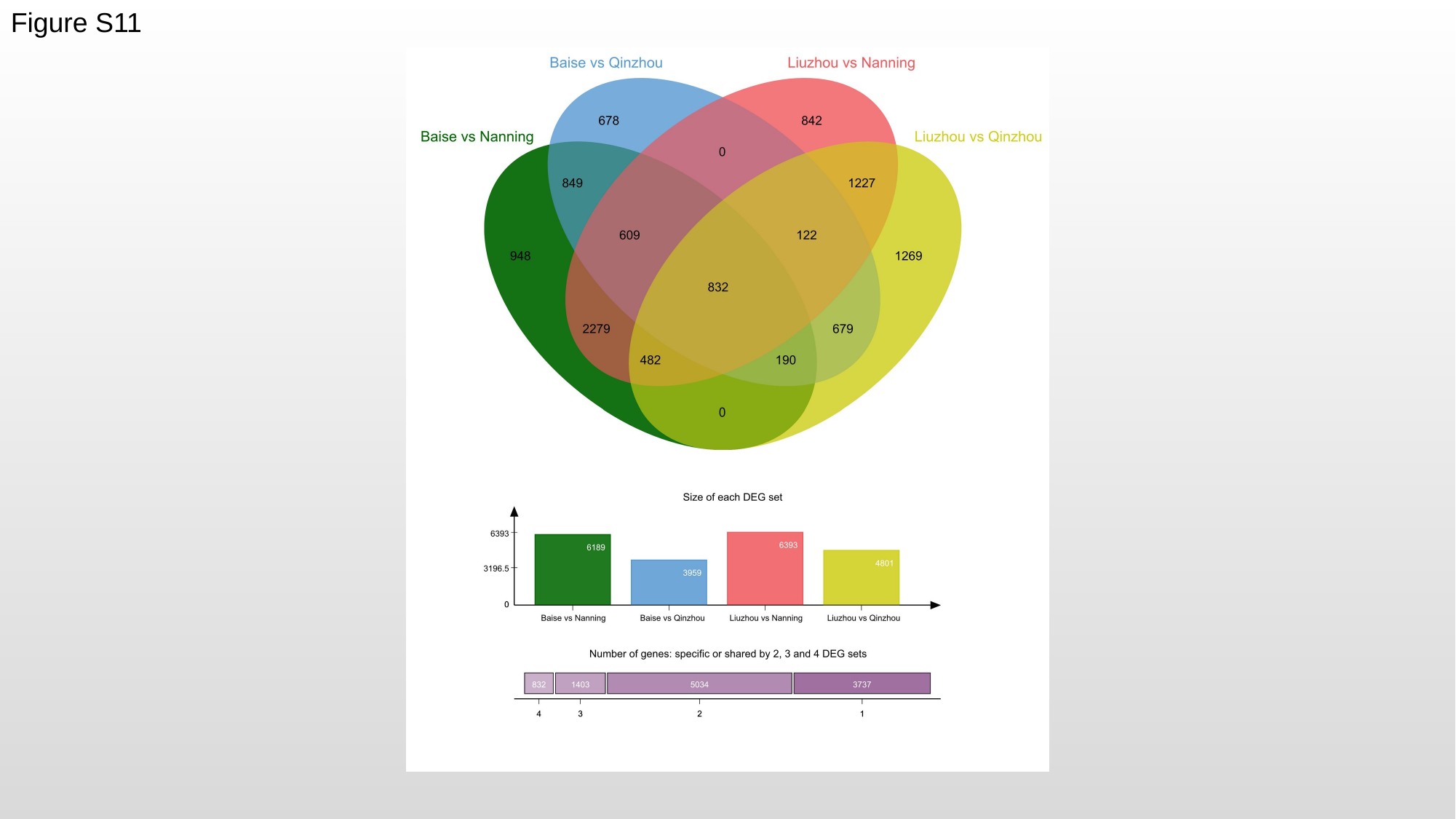

Figure S11

## Slide 12
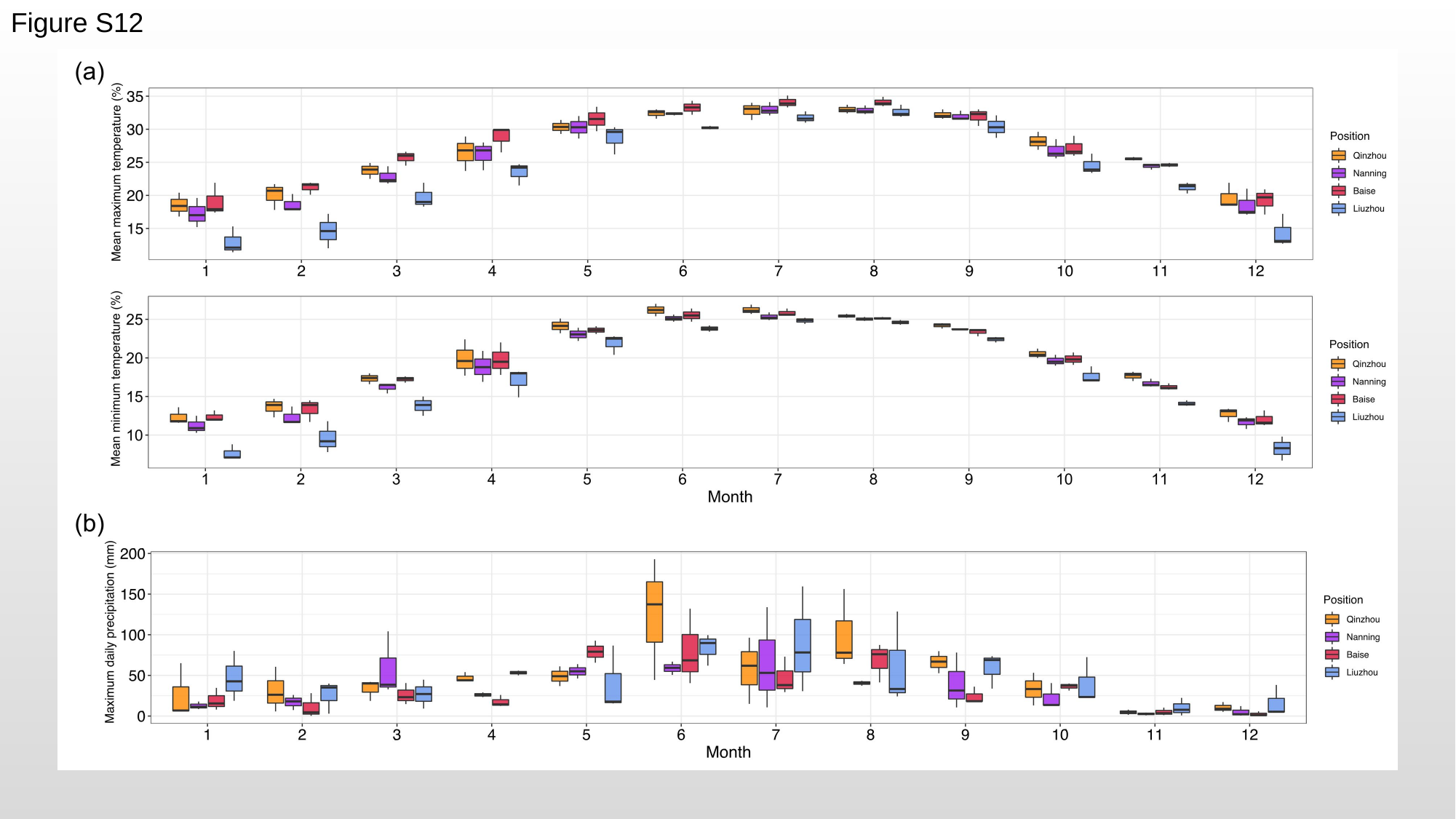

Figure S12
