# Supplementary material for: A Chromosome-Level Genome of the Camphor Tree and the Underlying Genetic and Climatic Factors for Its Top-Geoherbalism
Source: Front Plant Sci. 2022 Apr 21;13:827890. doi: 10.3389/fpls.2022.827890 (PMC9112071; doi:10.3389/fpls.2022.827890)
Supplement: Supplementary file 3 [file Data_Sheet_1.docx]

**Figure 1** The camphor tree and landscape of its genome. (a) Images of a camphor tree and its multiple tissues. (i) An ancient camphor tree, (ii) tissue-cultured seedings, (iii) seedings, (iv) young leaves, (v) flowers and (vi) fruits. (b) Circos plot of the *C. camphora* genome assembly. Circles from outside to inside: (i) chromosomes, (ii) *Gypsy* LTR density, (iii) *Copia* LTR density, (iii) total LTR density, (iv) gene density and (v) GC content. These density metrics were calculated with 1 Mb nonoverlapped sliding windows. The syntenic genomic blocks (>300 kb) are illustrated with orange lines.

**Figure 2** Phylogenetic analyses. The phylogenetic tree was constructed based on 172 single-copy orthologous genes of 16 species using two ANA-grade species as outgroups; node age and 95% confidence intervals are labeled. Pie charts show the proportions of gene families that underwent expansion or contraction. Predicted whole-genome duplication (WGD) events were only indicated for Laurales and Magnoliales.

**Figure 3** Gene duplication and evolution. (a) *Ks* distribution of paralogues in magnoliid species (*C. camphora*, *C. kanehirae*, *P. americana* and *L. chinense*) and orthologues between these magnoliids and *V. vinifera*. (b) Synteny blocks among *C. camphora*, *L. chinense* and *V. vinifera.* (c) *Ka*/*Ks* ratio distributions of gene pairs derived from different types of duplication. WGD, whole-genome duplication; TD, tandem duplication; PD, proximal duplication; TRD, transposed duplication; DSD, dispersed duplication. (d) *Ks* ratio distributions of gene pairs derived from different types of duplication. (e) GO enrichment analyses of genes from different types of duplication. The enriched GO terms with adjusted *P* values < 0.01 are presented. The colours of the bubbles indicate the statistical significance of the enriched GO terms. The sizes of the bubbles indicate the number of genes associated with one GO term. (f) KEGG enrichment analyses of genes resulting from different types of duplication. Enriched KEGG pathways with adjusted *P* values < 0.01 are presented. The colours of the bubbles represent the statistical significance of enriched KEGG pathways. The sizes of the bubbles indicate the number of genes associated with one KEGG pathway.

**Figure 4** Abundance patterns of volatile metabolites in *C. camphora* planted in different locations. (a) Pie charts show the proportions of different types of metabolites identified in the current study. (b) Hierarchical clustering heatmap of metabolic abundance profiles in the four planting locations, including Qinzhou, Nanning, Baise and Liuzhou, indicated on the x axis. Metabolic abundance was averaged and z-score transformed. The rows are clustered by the types of metabolites. (c) Principal component analyses (PCA) of metabolites of *C. camphora* planted in the four locations. The circles represent the 95% confidence intervals. (d) The relative abundances of linalool, borneol, camphor, 1,8-cineole and isonerolidol in the four planting locations.

**Figure 5** Genes involved in the biosynthesis of volatile terpenoids. (a) Phylogenetic analyses of *TPS* genes in *C. camphora*. The phylogenetic tree was constructed based on *TPS* gene sequences from four magnoliid genomes (*C. camphora*, *C. kanehirae*, *P. americana* and *L. chinense*) and *A. thaliana*. (b) Copy numbers of *TPS* genes in the genomes of four magnoliids and *A. thaliana*. (c) Distribution of the *TPS* genes on seven chromosomes of *C. camphora*. (d) Tissue-specific expressions of *TPS-a*, *TPS-b* and *TPS-g* subfamilies.

**Figure 6** Biosynthetic pathways of monoterpenoids and sesquiterpenoids. Relative expression profiling of genes involved in volatile terpenoid biosynthesis among the four planting locations (Qinzhou, Nanning, Baise and Liuzhou). Gene expression was extracted from the combined differentially expressed gene (DEG) set (Liuzhou vs. Qinzhou, Liuzhou vs. Nanning, Baise vs. Qinzhou and Baise vs. Nanning). MEP, mevalonate pathway; MEP, methylerythritol phosphate pathway; ACAT, acyl-coenzyme A-cholesterol acyl-transferase; HMGS, hydroxymethylglutaryl coenzyme A synthase; HMGR, hydroxymethylglutaryl coenzyme A reductase; MVK, mevalonate kinase; PMK, phospho-mevalonate kinase; MVD, mevalonate diphosphate decarboxylase; DXS, 1-deoxy-D-xylulose 5-phosphate synthase; DXR, 1-deoxy-D-xylulose 5-phosphate reductoisomerase; MCT, 2-C-methyl-D-erythritol-4-phosphate cytidylyltransferase; CMK, 4-(cytidine-5-diphospho)-2-C-methyl-D-erythritol kinase; MDS, 2-C-methyl-D-erythritol-2,4-cyclodiphosphate synthase; HDS, (E)-4-hydroxy-3-methyl-but-2-enyl-pyrophosphate synthase; HDR, (E)-4-hydroxy-3-methyl-but-2-enyl-pyrophosphate reductase.

**Figure 7** Analyses of climatic factors in different planting locations. (a) Principal component analyses (PCA) of seventeen climatic factors in the four planting locations in 2018, 2019 and 2020. (b) The loadings of climatic factors in the PCA plot. The colours of the arrows represent the percentages of the contributions of climatic factors to the PCs. (c) Histograms of the percentages of the contributions of different climatic factors to PC1. The red dashed lines indicate the average contributions of different climatic factors. Only the top eight climatic factors are shown. (d) Histograms of the percentages of the contributions of different climatic factors to PC2. The red dashed lines indicate the average contributions of different climatic factors. Only the top eight climatic factors are shown. (e) Monthly observations of the mean temperature in the four planting locations. Single and double asterisks indicate statistically significance levels of P < 0.05 and P < 0.01, respectively, between Liuzhou and Qinzhou/Nanning (paired-sample Student’s t test). (f) Monthly observations of daily precipitation in the four planting locations. A single asterisk indicates the statistically significance levels of P < 0.05 between Baise and Qinzhou/Nanning (paired-sample Student’s t test).

**Figure S1** Genome-wide Hi-C heatmap of *C. camphora*. Post-clustering heatmap shows density of Hi-C interactions between contigs from 3D-DNA pipeline.

**Figure S2** Analyses of LTR insertion time. (a) Frequency distribution of LTR insertion time in magnoliids and *V. vinifera*. (b) Frequency distribution of *Copia*, *Gypsy* and unknown LTR insertion time in *C. camphora*.

**Figure S3** Reconstruction for phylogenetic trees. (a) Concatenation-based phylogenetic tree constructed by 172 strictly single-copy orthologous genes retrieved from 16 plants using IQ-TREE. (b) Coalescent-based phylogenetic tree constructed by the 172 genes. RAxML was used for construction of the 172 gene trees. The coalescent-based species tree was finally reconstructed by ASTRAL-III.

**Figure S4** Intra-genomic synteny in *C. camphora.*

**Figure S5** Inter-genomic synteny among *C. camphora*, *L. chinense* and *V. vinifera.*

**Figure S6** GO enrichment analyses of different types of duplicate genes. The abbreviations see “Methods” section.

**Figure S7** KEGG enrichment analyses of different types of duplicate genes. The abbreviations see “Methods” section.

**Figure S8** The numbers of genes among different types of duplicate genes. The abbreviations see “Methods” section.

**Figure S9** Hierarchical clustering (a) and principal component analyses (b) of metabolic abundance for quality control.

**Figure S10** The relative abundance of terpenes, esters, ketones, heterocyclic compounds, alcohols and aromatics in four different planting locations.

**Figure S11** The venn diagram and bar plot of differentially expressed genes of “Liuzhou vs Qinzhou”, “Liuzhou vs Nanning”, “Baise vs Qinzhou” and “Baise vs Nanning”.

**Figure S12** The monthly observations of mean maximum temperature, mean minimum temperature and maximum daily precipitation in the four planting locations, including Qinzhou, Nanning, Baise and Liuzhou.
